# Colchicine Use in Acute Coronary Syndrome: A Systematic Review and Meta-Analysis

**DOI:** 10.3390/jcm15010105

**Published:** 2025-12-23

**Authors:** Huey Chiat Cheong, Meng Hsuan Kuo, Chih-Wei Tseng, Yi-Da Li

**Affiliations:** 1 Division of Cardiology, Department of Internal Medicine, Dalin Tzu Chi Hospital, Buddhist Tzu Chi Medical Foundation, No. 2, Minsheng Rd., Dalin Township, Chiayi County 62247, Taiwan; dl2465@tzuchi.com.tw; 2 School of Medicine, Tzu Chi University, Hualien City 970374, Taiwan; dm650504@tzuchi.com.tw; 3 Department of Pharmacy, Dalin Tzu Chi Hospital, Buddhist Tzu Chi Medical Foundation, Chiayi County 62247, Taiwan; df441865@tzuchi.com.tw; 4 Division of Gastroenterology, Department of Internal Medicine, Dalin Tzu Chi Hospital, Buddhist Tzu Chi Medical Foundation, Chiayi County 62247, Taiwan

**Keywords:** colchicine, acute coronary syndrome, inflammation, major adverse cardiovascular events, meta-analysis

## Abstract

**Background**: This study aimed to assess the efficacy, optimal dosing, and timing of colchicine therapy in reducing major adverse cardiovascular events (MACE), its impact on inflammatory markers, and safety concerns in patients following acute coronary syndrome (ACS) through a systematic review and meta-analysis of randomized controlled trials (RCTs). **Methods:** A comprehensive search of PubMed, Embase, and the Cochrane Library was conducted in accordance with PRISMA guidelines to identify RCTs comparing colchicine versus placebo or standard treatment in ACS patients. The primary outcome was MACE and secondary outcomes included all-cause and cardiovascular mortality, non-fatal MI, stroke, revascularization, heart failure, CRP/hs-CRP changes, and adverse effects. Fifteen RCTs involving 19,131 patients were analyzed. **Results:** The benefit of colchicine in reducing MACE risk was marginally significant (RR = 0.79, 95% CI: 0.63–0.99, *p* = 0.04, I^2^ = 59%). No significant reduction was observed for all-cause mortality, cardiovascular mortality, other cardiovascular outcomes, early initiation of colchicine (≤3 days), or choice of dosage (≤0.5 mg/day vs. >0.5 mg/day). The findings pertaining to the delayed time-to-initiation (>3 days) and changes in CRP or hs-CRP levels were inconclusive. Gastrointestinal side effects, especially diarrhea (RR = 1.76, 95% CI: 1.16–2.66, *p* = 0.001), were most common. No increase in hematologic events or infections was observed. **Conclusions:** Colchicine potentially reduces MACE in ACS patients, without evidence of benefit in improving all-cause mortality or other cardiovascular outcomes. Gastrointestinal intolerance is the most common side effect. This result is consistent with current clinical guidelines: a Class IIb recommendation for colchicine use in ACS. There is a need for further high-quality trials to refine patient selection and optimize treatment regimens.

## 1. Introduction

Inflammation is vital for myocardium recovery after acute coronary syndrome (ACS), but prolonged immune activation can exacerbate tissue damage [1,2]. High levels of inflammatory markers like C-reactive protein (CRP) were found to predict an unfavorable disease course during acute myocardial infarction (AMI) [3,4]. A previous anti-inflammatory drug trial targeting interleukin-1β in atherosclerotic disease observed a significant reduction in cardiovascular death, but at the expense of increased fatal infection, emphasizing the importance of balanced inflammatory regulation [5].

Colchicine is attractive as an anti-inflammatory medication due to its affordability, availability and relatively safe profile [6,7]. It was brought to attention when a retrospective, cross-sectional study showed lower myocardial infarction prevalence in gout patients using colchicine [8]. Subsequent randomized trials yielded mixed results when colchicine was used in patients with ACS. A significant reduction in major adverse cardiovascular events (MACE) was observed in the COLCOT study when colchicine was used within 30 days of AMI, and it was most efficacious when prescribed within the first three days [9,10]. Akrami et al. [11] supported the findings of this study. In contrast, the COVERT-MI [12], COPS [13], and Yousefzadeh et al. [14] trials failed to show significant benefits. Most recently, the CLEAR-SYNERGY (OASIS 9) trial (*N* = 7062) also reported no significant advantage of low-dose colchicine compared with placebo, even when administered within three days of AMI onset [15].

The conflicting findings surrounding colchicine’s therapeutic effects are apparent not only in its influence on MACE but also in its modulation of inflammatory biomarkers. CRP and high-sensitivity CRP (hs-CRP) were commonly assessed in prior investigations; however, results across these studies have remained inconsistent [14,16,17,18,19].

The observed inconsistencies in clinical outcomes may, in part, be attributable to variations in treatment regimens. The COLCOT trial demonstrated the greatest reduction in MACE when colchicine was administered within the first three days of AMI. However, this finding was not replicated in the CLEAR-SYNERGY (OASIS 9) trial. Across studies, colchicine regimens have varied considerably, with some trials requiring a loading dose [14,20,21], while others omitted it, followed by either a low-dose (0.5 mg once daily) [9,10,11,14] or high-dose maintenance regimen (1.0 mg once daily or 0.5 mg twice daily) [12,13]. Furthermore, discrepancies in time-to-treatment initiation [10,17,18] and the duration of use [9,12,16] may further complicate interpretation. These variations underscore the need for subgroup meta-analyses to clarify the optimal regimen and inform evidence-based prescribing strategies.

Despite conflicting trial results, previous meta-analyses have supported the use of colchicine in patients with coronary artery disease [22,23,24,25]. The American Heart Association (AHA) guideline provided a Class IIb recommendation on long-term use of low-dose colchicine to reduce the risk of MACE after ACS [26], while the European Society of Cardiology (ESC) guideline gives a Class IIb recommendation for ACS [27] and a Class IIa for chronic coronary syndrome [28]. The results of the CLEAR-SYNERGY (OASIS 9) trial [15] offer a timely opportunity for an updated systematic review and meta-analysis to clarify colchicine’s therapeutic role and optimal regimen in ACS. This study aimed to provide an updated assessment of its efficacy and dosing strategies.

## 2. Materials and Methods

This systematic review and meta-analysis was pre-registered on PROSPERO (ID number: CRD420251041546) and followed the Preferred Reporting Items for Systematic Reviews and Meta-Analyses (PRISMA) guidelines (Table S1) [29].

### 2.1. Search Strategy

A comprehensive search was conducted in PubMed, Embase and the Cochrane Library from database inception to 23 January 2025, with no language restrictions, to identify relevant RCTs (randomized controlled trials). The search strategy was developed by two researchers (CWT and MHK) (Table S2). The key search terms included “acute coronary syndrome”, “acute myocardial infarction” and “colchicine”, incorporating Medical Subject Headings (MeSH), where applicable. Additional articles were identified in the reference lists of pertinent original studies and relevant reviews.

### 2.2. Inclusion Criteria

After removing duplicate records, two reviewers (YDL and HCC) independently selected the included studies based on the following parameters: (1) Patients: Adults with acute coronary syndrome; (2) Exposures: colchicine; (3) Comparison: placebo or standard treatment; (4) Outcome: MACE (cardiovascular mortality, non-fatal myocardial infarction, stroke, angina requiring revascularization, and heart failure), all-cause mortality, inflammatory level change (CRP and hs-CRP), and adverse effects (gastrointestinal side effects, hematologic events (myelotoxicity or myelosuppression, such as anemia, leukopenia, thrombocytopenia or pancytopenia), and infections); (5) Study design: RCTs only.

### 2.3. Literature Selection and Data Extraction

Two reviewers (YDL and HCC) individually screened titles and abstracts based on the inclusion criteria and read the full-text articles for final eligibility. A third reviewer (CWT) was consulted to resolve any discrepancies in the study selection.

Two authors (YDL and HCC) independently collected data, including the name of the first author, publication year, country, study population, number of patients, treatment regimens (dose, duration, duration of intervention, and time to first colchicine intervention), follow-up period, age, sex ratio, comorbidity, co-medication, and statistical data on the influence of safety and adverse events. For continuous variables, means and standard deviations (SDs) were extracted. If SDs for changes from baseline were not reported, they were imputed using a correlation coefficient approach [30]. Any discrepancies in data extraction were resolved by discussion. Two study cohorts were found to report on two different outcome measures in different publications. Only the required outcome data were extracted from each of the publications, while taking caution to avoid duplicates during analysis (COVERT-MI study: Data related to MACE from Bouleti et al. [20], CRP and left ventricular thrombus from Mewton et al. [12]; COLCOT study: Data related to time to first intervention and corresponding MACE from Bouabdallaoui et al. [10], and adverse effects from Tardif et al. [9]).

### 2.4. Risk of Bias Assessment

The methodological quality of included RCTs was assessed by two researchers (YDL and MHK) independently. We used the Cochrane Collaboration’s ROB tool 2.0 [31], addressing the critical domains of randomization process, deviations from intended interventions, missing outcome data, measurement of the outcome, selection of the reported result and overall bias, to evaluate the methodological quality of the included RCTs.

### 2.5. Data Synthesis and Statistical Analysis

We conducted a random-effects meta-analysis due to the expected clinical heterogeneity among the included RCTs. Statistical heterogeneity across the included studies was quantified using the I^2^ statistic, with substantial heterogeneity defined as I^2^ > 50 [30]. Subgroup analyses performed included colchicine intervention time (0–3 days, 4–7 days, and ≥8 days) in accordance with the COLCOT study design [10] and colchicine dosage (low dose: ≤0.5 mg/day; high dose: >0.5 mg/day). Publication bias was evaluated by assessing funnel plot asymmetry for meta-analyses of outcomes that included ≥10 studies [32]. To assess the robustness of the results of our main analyses, we conducted a sensitivity analysis using the leave-one-out meta-analysis. A two-sided *p* < 0.05 was considered statistically significant, and all analyses were performed using Comprehensive Meta-Analysis software (version 4.0, Biostat, Englewood, NJ, USA) and Review Manager Version 5.3 (Cochrane Collaboration, 2020).

### 2.6. Certainty of Evidence of the Study Outcomes

Two independent reviewers (MHK and HCC) evaluated the CoE for each study outcome based on the Grading of Recommendations Assessment, Development and Evaluation (GRADE) criteria [33]. Any discrepancy between the review authors was resolved by discussion with the senior author (CWT).

The datasets generated and analyzed during this current study are available from the corresponding author upon reasonable request via E-mail.

## 3. Results

### 3.1. Literature Search and Study Selection

**Figure 1:**
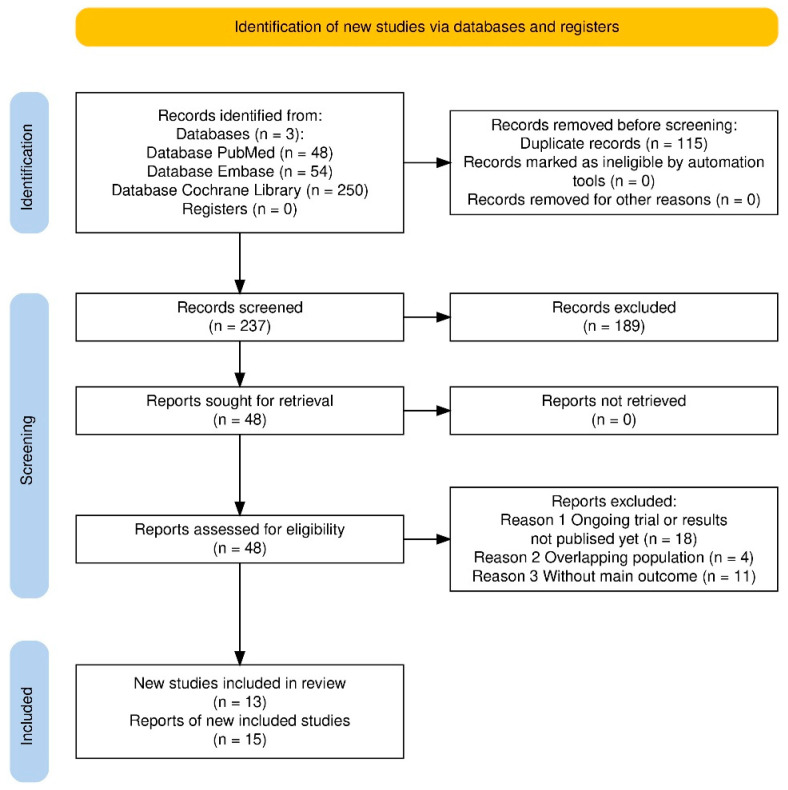
PRISMA 2020 flowchart of the study selection process.

A total of 352 records were identified through database searches (PubMed: 48, Embase: 54, Cochrane Library: 250). After removing 115 duplicate records, 237 studies were screened based on titles and abstracts. Following the exclusion of 189 ineligible studies, 48 full-text articles were assessed for eligibility. Of these, 33 studies were excluded due to ongoing trials or lack of published results (*n* = 18), overlapping study populations (*n* = 4), and lack of primary outcome reporting (*n* = 11). Ultimately, 15 RCT reports were included in this meta-analysis, and were all written in English. The PRISMA flow diagram illustrating the study selection process is provided in Figure 1.

### 3.2. Characteristics of Included Studies

The meta-analysis included 15 RCT reports, comprising a total of 19,131 patients with ACS. The study populations varied in terms of geographic location, baseline characteristics, colchicine dosing regimens, and follow-up duration (Table 1). The majority of studies enrolled patients with ST-elevation myocardial infarction (STEMI), non-ST-elevation myocardial infarction (NSTEMI), or unstable angina (UA), with sample sizes ranging from 44 to 7062 participants. The duration of follow-ups ranged from 30 days to 2.98 years.

The baseline characteristics of the study populations were generally comparable, with mean ages ranging from 54.5 to 61 years, and predominantly male participants. The prevalence of key cardiovascular risk factors varied, with smoking (30–72%), hypertension (31–51%), diabetes (13–49%), and dyslipidemia (13–52%) reported across the studies. Further details on study characteristics, including comorbidities and concurrent cardiovascular therapies, are provided in Table 1.

### 3.3. Risk of Bias of Included Studies

**Figure 2:**
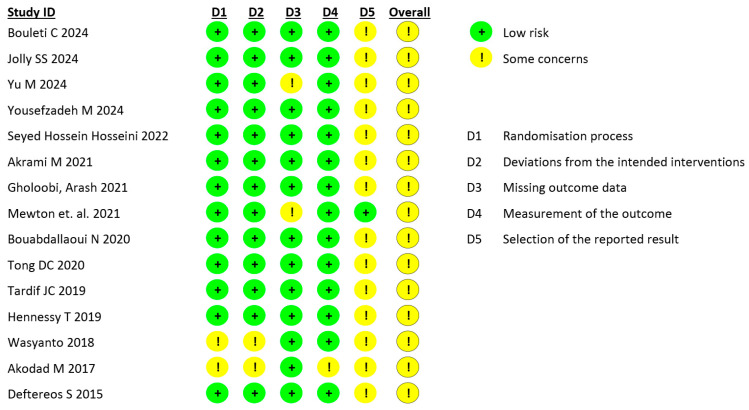
Quality assessment of the included trials [9,10,11,12,13,14,15,16,17,18,19,20,21,34,35].

All included studies were assessed as having some concerns in the overall risk of bias evaluation. The primary source of bias was the potential for selective reporting of results (Figure 2).

### 3.4. Primary Outcome: MACE and Subgroup Analyses

**Figure 3:**
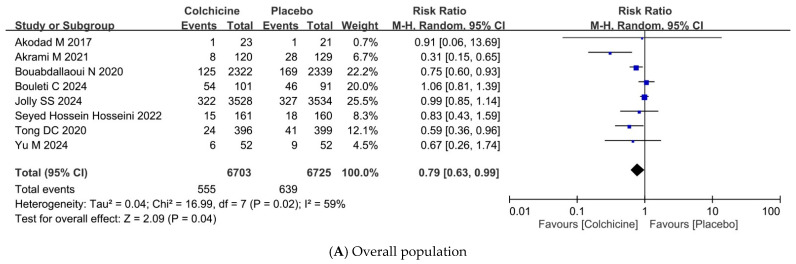

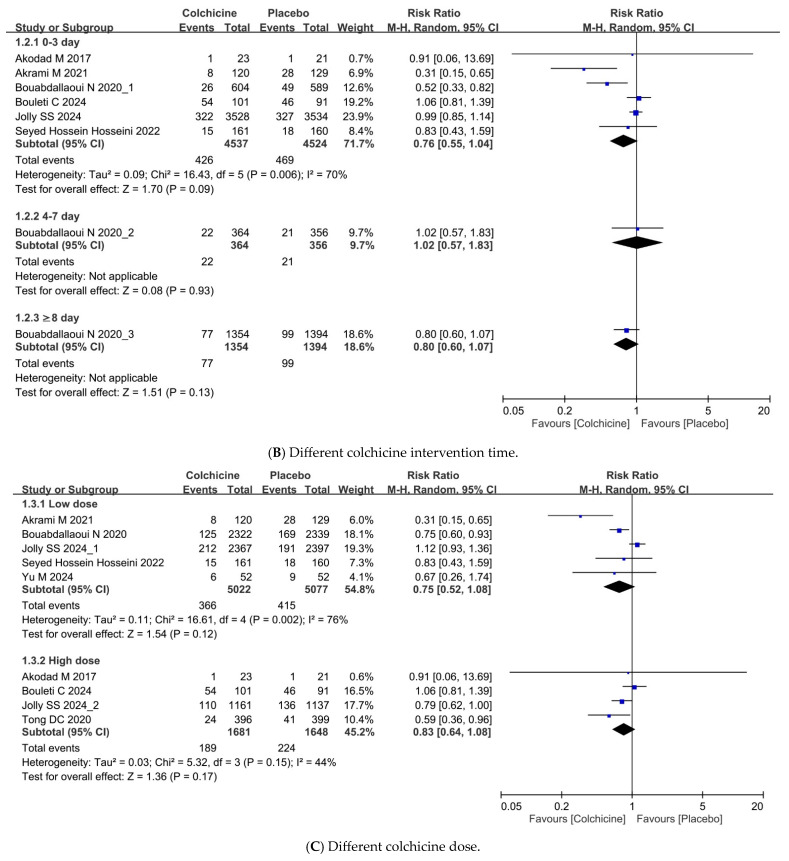
Forest plots showing summary risk ratio and 95% CI for MACE. MACE, Major Adverse Cardiovascular Events [10,11,13,15,17,20,34,35].

Among the eight RCTs with reporting MACE, comprising 13,428 patients, colchicine reduced the risk of MACE compared to placebo or standard treatment at a marginal statistical significance (RR = 0.79, 95% CI: 0.63–0.99, *p* = 0.04) (Figure 3A, Table 2). However, moderate heterogeneity (I^2^ = 59%) was observed across studies, suggesting some variability in treatment effects among different populations and study designs.

Subgroup analyses based on the timing of colchicine initiation did not reveal a clear benefit. Initiation within 0–3 days post-ACS (RR = 0.76, 95% CI: 0.55–1.04, *p* = 0.09, I^2^ = 70%), 4–7 days post-ACS (RR = 1.02, 95% CI: 0.57–1.83, *p* = 0.93), and ≥8 days post-ACS (RR = 0.80, 95% CI: 0.60–1.07, *p* = 0.13) showed no significant reduction in MACE risk (Figure 3B). Similarly, neither low-dose colchicine (≤0.5 mg/day) (RR = 0.75, 95% CI: 0.52–1.08, *p* = 0.12, I^2^ = 76%) nor high-dose colchicine (>0.5 mg/day) (RR = 0.83, 95% CI: 0.64–1.08, *p* = 0.17, I^2^ = 44%) significantly reduced MACE (Figure 3C).

### 3.5. Secondary Outcomes: All-Cause Mortality, Cardiovascular Mortality, Non-Fatal Myocardial Infarction, Stroke, Angina Requiring Revascularization, Heart Failure, Crp Change, Hs-Crp Change

Colchicine treatment did not demonstrate a significant effect on mortality outcomes (Table 2). The pooled analysis of nine RCTs including 13,548 patients showed no significant reduction in all-cause mortality (RR = 0.94, 95% CI: 0.78–1.12, *p* = 0.45, I^2^ = 0%), cardiovascular mortality (RR = 1.03, 95% CI: 0.81–1.29, *p* = 0.51, I^2^ = 0%), or non-fatal myocardial infarction (RR = 0.85, 95% CI: 0.69–1.05, *p* = 0.38, I^2^ = 6%), suggesting that colchicine does not confer a survival benefit in ACS patients. Stroke risk was lower in the colchicine group but did not reach significance (RR = 0.62, 95% CI: 0.28–1.36, *p* = 0.15, I^2^ = 39%). Similarly, angina requiring revascularization (RR = 0.73, 95% CI: 0.45–1.20, *p* = 0.09, I^2^ = 53%) and heart failure incidence (RR = 0.96, 95% CI: 0.49–1.89, *p* = 0.21, I^2^ = 35%) remained unaffected by colchicine treatment.

As for the changes in inflammatory markers CRP (mean difference = 3.43, 95% CI: −13.38 to 20.24, *p* = 0.45, I^2^ = 99%) and hs-CRP (mean difference = −0.43, 95% CI: −1.28 to 0.42, *p* = 0.32, I^2^ = 90%), the result was not interpretable due to high heterogeneity (Supplementary Figure S2). The high heterogeneity observed suggests substantial variability among studies in terms of baseline inflammatory status, colchicine dosing, and treatment duration.

### 3.6. Safety Outcome

Colchicine treatment was associated with an increased risk of gastrointestinal (GI) adverse events, particularly diarrhea, but did not show a significant impact on hematologic events or infections. In the pooled analysis of 10 studies with 13,693 patients, colchicine significantly increased the incidence of GI events, including all GI symptoms and serious events (RR = 1.49, 95% CI: 1.10–2.01, *p* < 0.001, I^2^ = 76%) (Table 3). The most frequently reported symptom was diarrhea, with colchicine users experiencing a 76% increased risk compared to controls (RR = 1.76, 95% CI: 1.16–2.66, *p* = 0.001, I^2^ = 76%). The high heterogeneity suggests variability in study populations and treatment durations, but the overall trend indicates that colchicine use is associated with notable GI intolerance. Colchicine did not significantly increase the risk of hematologic events (RR = 0.59, 95% CI: 0.21–1.67, *p* = 0.24, I^2^ = 27%) or infections (RR = 1.10, 95% CI: 0.72–1.68, *p* = 0.15, I^2^ = 41%).

### 3.7. Publication Bias and Sensitivity Analysis

**Figure 4:**
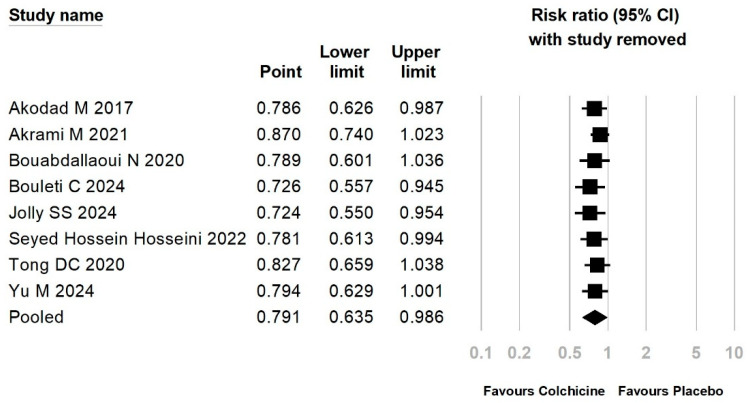
Sensitivity analysis for MACEs [10,11,13,15,17,20,34,35].

The distribution of reports on MACE, as shown in the funnel plot, exhibits a symmetrical pattern, suggesting the absence of publication bias (*p* = 0.07; Egger’s test) (Supplementary Figure S1). Sensitivity analysis using a leave-one-out approach demonstrated that the overall estimates for MACE and thrombosis events remained largely unchanged, indicating that no single study had a disproportionate influence on the pooled results (Figure 4).

### 3.8. GRADE Assessment

We judged the CoE by the GRADE criteria for our primary and secondary outcomes to be low to high (Supplementary Table S3).

## 4. Discussion

Our meta-analysis demonstrates that colchicine provides a borderline-significant reduction in MACE among ACS patients. There were no significant differences in all-cause mortality, cardiovascular mortality, non-fatal MI, stroke, angina requiring revascularization, and heart failure. Early initiation (<3 days) and different dosing strategies (≤0.5 mg/day vs. >0.5 mg/day) did not yield meaningful differences in outcomes. Findings for delayed time-to-initiation (≥3 days) or for changes in CRP or hs-CRP were inconclusive. Gastrointestinal side effects were more common with colchicine, potentially impacting adherence.

Although the CLEAR SYNERGY trial [15]—a large, multicenter study enrolling over 7000 patients with AMI—did not demonstrate a significant reduction in MACE with colchicine therapy, our meta-analysis, which incorporated this trial, continued to show benefits for patients with ACS. The pooled analysis of over 13,000 patients revealed a reduction in MACE (RR: 0.79, 95% CI: 0.63–0.99, *p* = 0.04), but with moderate heterogeneity (I^2^ = 59%). Two potential factors may account for this discrepancy. First, the variations in trial designs, patient characteristics, treatment protocols, and follow-up duration may have influenced the results. Furthermore, the definition of MACE varies among the eight RCTs (Supplementary Tables S4 and S5). This worsened heterogeneity and made our findings less robust than anticipated. Second, sensitivity analyses identified four trials having a substantial impact on the overall estimates [10,11,13,34]. Notably, the COLCOT subgroup [10] was the largest positive contributor due to its sample size. Additionally, the trials by Akrami et al. [11] and Yu et al. [34] included a relatively high number of unstable angina cases within their composite MACE definitions. The reduction in MACE observed in the Akrami et al. [11] trial was primarily attributable to a lower incidence of unstable angina, raising the possibility that colchicine may reduce the occurrence of post-infarction unstable angina. The inclusion of such data has contributed to a slightly favorable outcome seen in our analysis, and the removal of these four trials easily shifts the results to non-significance. As such, we recommend cautious interpretation of this statistical outcome. The significance would be more pronounced if there were more participants/trials included. Nonetheless, our findings are consistent with the current clinical guidelines: a Class IIb recommendation for colchicine use in ACS patients. Further high-quality studies are warranted to refine the target population and to determine which specific components of MACE derive the greatest clinical benefit.

The timing of colchicine initiation has been proposed as a critical determinant of its therapeutic efficacy. A sub-analysis of the COLCOT trial [10] reported that initiating colchicine within three days of myocardial infarction significantly reduced MACE compared to placebo (HR: 0.52; 95% CI: 0.32–0.84). However, our subgroup analysis, where most of the included trials initiated colchicine within 0–3 days, did not demonstrate statistically significant benefit (RR = 0.76, 95% CI = 0.55–1.04, *p* = 0.09, I^2^ = 70%). One reason is that the small sample size within each subgroup may not be sensitive enough to detect a significant difference. The other reason was the substantial heterogeneity across trials (differences in patient characteristics, study designs, and colchicine protocols), probably obscuring any potential timing effect. Interpretation of the subgroups with delayed colchicine initiation (within 4–7 days and ≥ 8 days) was limited because each was informed by only a single study. In brief, early colchicine initiation did not significantly reduce MACE, and the evidence is insufficient to confirm or refute any benefit from delayed initiation in ACS patients. Future well-powered studies specifically designed to evaluate the impact of initiation timing are warranted.

Dosing strategy has also been hypothesized to influence the efficacy of colchicine. A subgroup analysis from the CLEAR SYNERGY trial suggested a potential benefit with high-dose colchicine (0.5 mg twice daily) [15], but our meta-analysis did not reveal such an advantage. Neither low-dose colchicine (≤0.5 mg/day) (RR = 0.75, 95% CI: 0.52–1.08, *p* = 0.12, I^2^ = 76%) nor high-dose colchicine (>0.5 mg/day) (RR = 0.83, 95% CI: 0.64–1.08, *p* = 0.17, I^2^ = 44%) significantly reduced MACE. The differences in patient baseline characteristics, such as comorbidities and background therapies, may also influence individual responses, obscuring potential dose-related effects. While higher doses may provide greater anti-inflammatory effects, the effect may not be sufficient to translate into a clinically meaningful reduction in cardiovascular events. Furthermore, higher doses of colchicine are associated with a raised incidence of gastrointestinal adverse events, contributing to reduced medication adherence and attenuated clinical benefit. Overall, increasing colchicine dosage may not offer additional clinical benefit and instead, may increase the risk of adverse outcomes.

Our meta-analysis found no significant reduction in CRP or hs-CRP with colchicine use in ACS patients, consistent with prior analyses [24,36]; however, the extreme heterogeneity makes this result uninterpretable. While some trials reported reductions in these inflammatory markers [16,19], these findings may be confounded by differences in baseline inflammatory marker levels between groups and the inconsistent measurement timing. The lack of standardization has limited the meaningful interpretation of our findings. Concurrently, there are other factors that could contribute to the lack of meaningful changes in the inflammatory markers. Chronic low-grade inflammation from comorbidities may attenuate observable changes in CRP/hs-CRP. Traditional inflammatory markers like CRP may not fully capture colchicine’s anti-inflammatory impact, highlighting the need for studies using alternative biomarkers such as MMP-9, NOX2, and TGF-β1 [37], which may more accurately reflect colchicine’s anti-inflammatory effects in future studies.

Colchicine has a narrow therapeutic window. It primarily causes toxicity by disrupting cell division, especially in rapidly proliferating tissues like the gastrointestinal lining and bone marrow [6,7]. Our meta-analysis confirmed a significantly increased risk of gastrointestinal side effects, especially diarrhea, even when low-dose colchicine is used, consistent with prior meta-analyses [23,38]. Fortunately, serious adverse events such as hematological events or infections were not significantly elevated. Interestingly, one study [12] raised the issue of a potential association between colchicine and left ventricular thrombus formation, a finding that has not been consistently observed in the past [6,38]. While there were rare instances of venous thromboembolism [9,14], further investigation is required to clarify any prothrombotic risk associated with colchicine use.

This meta-analysis has several strengths, mainly by including 15 RCTs with over 19,000 patients and performing multiple subgroup analyses in an attempt to identify potential subgroups that may benefit from colchicine use. Additionally, our methodological approach, particularly the use of mean absolute change for inflammatory biomarker analysis, ensures a more accurate assessment of colchicine’s impact on systemic inflammation. However, we acknowledge several limitations. The inconsistent definition of MACE, the limited number of studies available for subgroup analysis, and the moderate to high heterogeneity made outcome interpretation difficult. In addition, most studies lacked long-term follow-up, limiting conclusions about sustained benefits of colchicine in secondary prevention. These limitations underscore the need for a well-designed study to clarify colchicine’s role in ACS.

## 5. Conclusions

The benefit of colchicine in ACS patients for reducing MACE is of marginal statistical significance and should be interpreted with caution due to moderate heterogeneity and sensitivity to the inclusion of certain studies. While gastrointestinal side effects are common, serious adverse events are rare, though potential thrombotic risks will require further investigation. Given these findings, colchicine may still be useful as an adjunctive therapy and could offer some cardiovascular benefits in selected ACS populations. Future large-scale trials with standardized endpoints, longer follow-up, and more sensitive inflammatory biomarkers are warranted to fully clarify its therapeutic role.

## Figures and Tables

**Table 1:** Demographic data and characteristics of included RCTs.

Author (Year)	Location	Study Population (*N*)	Parameters	Colchicine Dosage/ Duration	Duration of Intervention	Time to Colchicine First Intervention	Age (Mean, SD)	Sex M/F	Follow Up	Smoking (*n*, %)	Hypertension (*n*, %)	Diabetes (*n*, %)	Dyslipidemia (*n*, %)
Bouleti C (2024) [20]	France	STEMI (192)	C/P	LD 2 mg; MD 0.5 mg BID	5 days	At the time of revascularization	59.0 ± 10.6	154/38	12 months	83 (43%)	59 (31%)	25 (13%)	63 (33%)
Jolly SS (2024) [15]	Multi-country	STEMI/NSTEMI (6713/349)	C/P	BW ≥ 70 kg: 0.5 mg BID 90 days → 0.5 mg QD; BW ≤ 70 kg: 0.5 mg QD	Until end of trial	26.8 h	60.6 ± 10.3	5624/1438	2.98 years	2884 (41%)	3233 (46%)	1303 (18%)	NA
Yu M (2024) [34]	China	STEMI/NSTEMI/UA (5/34/89)	C/P	0.5 mg QD	12 months	0–3 days; 4–7 days; ≥8 days	55.7 ± 10.6	96/32	12 months	50 (39%)	80 (63%)	32 (25%)	56 (44%)
Yousefzadeh M (2024) [14]	Iran	STEMI (172)	C/P	LD: 2 mg; MD: 0.5 mg QD	30 days	NA	59.26 ± 7.92	114/58	40 days	NA	NA	NA	NA
Hosseini SH (2022) [35]	Iran	STEMI (321) ^b^	C/P	1 mg before PCI and 0.5 mg daily post-PCI	until discharge	before PCI	58.74 ± 10.39	254/67	NA	137 (43%)	127 (40%)	114 (36%)	67 (21%)
Akrami M (2021) [11]	Iran	STEMI/NSTEMI/UA (128/35/86)	C/P	0.5 mg QD	6 months	NA	56.9 ± 7.56	173/76	6 months	101 (41%)	111 (45%)	59 (24%)	73 (29%)
Gholoobi A (2021) [16]	Iran	STEMI (150)	C/P	BW ≥ 75 kg: 0.5 mg BID; BW ≤ 75 kg or CrCl < 50 mL/min: 0.5 mg QD	30 days	NA	60.87 ± 7.9	78/72	30 days	NA	NA	74 (49%)	NA
Mewton N (2021) [12]	France	STEMI (192)	C/P	LD: 2 mg; MD: 0.5 mg BID	5 days	20 min	59.0 ± 10.6	154/38	12 months	83 (43%)	59 (31%)	25 (13%)	63 (33%)
Bouabdallaoui N (2020) [10]	Multi-country	NA (4661)	C/P	0.5 mg QD	22.7 months ^a^	0–3 days; 4–7 days; ≥8 days	60.6 ± 10.6	3774/887	22.7 months ^a^	1382 (30%)	2377 (51%)	942 (20%)	NA
Tong DC (2020) [13]	Australia	STEMI/ NSTEMI/ UA/NA (390/357/26/22)	C/P	0.5 mg BID for 1 month → 0.5 mg QD	12 months	NA	59.7 ± 10.2	632/163	400 days	277 (35%)	400 (50%)	151 (19%)	365 (46%)
Tardif JC (2019) [9]	Multi-country	NA (4745)	C/P	0.5 mg QD	22.6 months ^a^	13.4 ± 10.2 days	60.6 ± 10.7	3836/909	22.6 months ^a^	1416 (30%)	2421 (51%)	959 (20%)	NA
Hennessy T (2019) [18]	Australia	STEMI (237)	C/P	0.5 mg QD	30 days	Within 7 days	61 ± 13.6	182/55	30 days	143 (60%)	112 (47%)	52 (22%)	NA
Wasyanto T (2018) [19]	Indonesia	STEMI/NSTEMI (23/9)	C/P	0.5 mg QD	5 days	NA	57.8	28/4	NA	23 (72%)	15 (47%)	7 (22%)	4 (13%)
Akodad M (2017) [17]	France	STEMI (44)	C/P	1 mg QD	30 days	First day of AMI	60.1 ± 13.1	35/9	30 days	31 (70%)	19 (43%)	6 (14%)	16 (36%)
Deftereos S (2015) [21]	Greece	STEMI (151)	C/P	LD: 2 mg; MD: 0.5 mg BID	5 days	NA	58	104/47	NA	79 (52%)	60 (40%)	32 (21%)	79 (52%)

^a^ median. ^b^ Only patients who received successful PCI were included. BW, body weight; C, Colchicine; CrCl, Creatinine clearance rate; LD, loading dose; MD, maintenance dose; NA, not available; NSTEMI, non-ST elevation myocardial infraction; P, Placebo; PCI, Percutaneous Coronary Intervention; STEMI, ST elevation myocardial infraction; UA, unstable angina.

**Table 2:** Summary risk ratio and 95% CI for primary and secondary outcomes.

Outcome	Study (*N*)	Patients (*N*)	RR	95% CI	*p*-Value	I^2^ (%)
MACE	8	13,428	0.79	0.63–0.99	0.04	59
Colchicine intervention time						
0–3 days	6	9061	0.76	0.55–1.04	0.09	70
4–7 days	1	720	1.02	0.57–1.83	---	---
≥8 days	1	2748	0.80	0.60–1.07	---	---
Colchicine Dose ^a^						
Low dose	5	10,099	0.75	0.52–1.08	0.12	76
High dose	4	3329	0.83	0.64–1.08	0.15	44
All-cause Mortality	9	13,548	0.93	0.78–1.12	0.45	0
Cardiovascular mortality	8	13,003	1.02	0.81–1.29	0.51	0
Non-fatal MI	9	13,087	0.85	0.69–1.05	0.38	6
Stroke	8	11,073	0.62	0.28–1.36	0.15	39
Angina requiring revascularization	4	10,709	0.73	0.45–1.20	0.09	53
Heart failure	4	5161	0.96	0.49–1.89	0.21	35

^a^ Low dose: 0.5 mg daily for a maximum of 1 month; High dose: >0.5 mg daily for maximum of 1 month. MACE, Major Adverse Cardiovascular Events; MI, myocardial infarction; RR, relative risk.

**Table 3:** Summary risk ratio and 95% CI for adverse effects.

Outcome	Records (*N*)	Patients (*N*)	RR	95% CI	*p*-Value	I^2^ (%)
GI events inclusive of all GI symptoms & serious events	10	13,693	1.49	1.10–2.01	<0.001	76
Diarrhea	6	12,438	1.76	1.16–2.66	0.001	76
Hematologic events regardless of severity	7	13,176	0.59	0.21–1.67	0.24	27
Infection events regardless of severity	5	12,230	1.10	0.72–1.68	0.15	41

CI, confidence interval; GI, gastrointestinal; RR, relative risk.

## Data Availability

The raw data supporting the conclusions of this article will be made available by the authors on request.

## Supplementary Materials

The following supporting information can be downloaded at: https://www.mdpi.com/article/10.3390/jcm15010105/s1, Table S1: PRISMA checklist; Table S2: Search strategy; Table S3: Grading of recommendations assessment, development, and evaluation (GRADE) summary of findings; Table S4: Definitions of MACE for each of the 8 RCTs included in the MACE analysis; Table S5: Comparison of MACE definitions for each of the 8 RCTs included in the MACE analysis; Figure S1: Funnel plot analysis of publication bias for MACE; Figure S2: Forest plot showing mean difference and 95% CI for CRP (A) and hs-CRP (B).

## Abbreviations

The following abbreviations are used in this manuscript:

ACS	Acute Coronary Syndrome
AMI	Acute Myocardial Infarction
CLEAR-SYNERGY	Colchicine and Spironolactone in Patients With ST-Elevation Myocardial Infarction (OASIS 9 trial)
COLCOT	Colchicine Cardiovascular Outcomes Trial
COPS	Colchicine in Patients with Acute Coronary Syndrome
COVERT-MI	Colchicine for Left Ventricular Infarct Size Treatment in Acute Myocardial Infarction
CRP/hs-CRP	C-Reactive Protein/high-sensitivity C-Reactive Protein
MACE	Major Adverse Cardiovascular Events
MMP-9	Matrix Metallopeptidase 9
NOX2	NADPH Oxidase 2
TGF-β1	Transforming Growth Factor Beta 1
