# Supplementary material for: Colchicine Use in Acute Coronary Syndrome: A Systematic Review and Meta-Analysis
Source: J Clin Med. 2025 Dec 23;15(1):105. doi: 10.3390/jcm15010105 (PMC12786563; doi:10.3390/jcm15010105)
Supplement: Supplementary file 1 [file jcm-15-00105-s001.zip › jcm-4009838-supplementary.pdf]

Supplementary Materials

Table S1 - PRISMA checklist

Table S2 - Search strategy

Table S3 - Grading of recommendations assessment, development, and evaluation (GRADE) summary of findings.

Table S4 - Definitions of MACE for each of the 8 RCTs included in the MACE analysis

Table S5 - Comparison of MACE definition for each of the 8 RCTs included in the MACE analysis

Figure S1. Funnel plot analysis of publication bias for MACE.

Figure S2. Forest plot showing mean difference (MD) and 95 % CI for CRP (A) and hs-CRP (B).

Table S1. PRISMA checklist

Section/topic	#	Checklist item	Reported on page #
**TITLE**			
Title	1	Identify the report as a systematic review, meta-analysis, or both.	Title page (p.1)
**ABSTRACT**			
Structured summary	2	Provide a structured summary including, as applicable: background; objectives; data sources; study eligibility criteria, participants, and interventions; study appraisal and synthesis methods; results; limitations; conclusions and implications of key findings; systematic review registration number.	p.1
**INTRODUCTION**			
Rationale	3	Describe the rationale for the review in the context of what is already known.	p.2
Objectives	4	Provide an explicit statement of questions being addressed with reference to participants, interventions, comparisons, outcomes, and study design (PICOS).	p.2
**METHODS**			
Protocol and registration	5	Indicate if a review protocol exists, if and where it can be accessed (e.g., Web address), and, if available, provide registration information including registration number.	p.3
Eligibility criteria	6	Specify study characteristics (e.g., PICOS, length of follow-up) and report characteristics (e.g., years considered, language, publication status) used as criteria for eligibility, giving rationale.	p.3
Information sources	7	Describe all information sources (e.g., databases with dates of coverage, contact with study authors to identify additional studies) in the search and date last searched.	p.3
Search	8	Present full electronic search strategy for at least one database, including any limits used, such that it could be repeated.	Supplementary Table S2

Study selection	9	State the process for selecting studies (i.e., screening, eligibility, included in systematic review, and, if applicable, included in the meta-analysis).	p.3
Data collection process	10	Describe method of data extraction from reports (e.g., piloted forms, independently, in duplicate) and any processes for obtaining and confirming data from investigators.	p.3
Data items	11	List and define all variables for which data were sought (e.g., PICOS, funding sources) and any assumptions and simplifications made.	p.3
Risk of bias in individual studies	12	Describe methods used for assessing risk of bias of individual studies (including specification of whether this was done at the study or outcome level), and how this information is to be used in any data synthesis.	p.3
Summary measures	13	State the principal summary measures (e.g., risk ratio, difference in means).	p.4
Synthesis of results	14	Describe the methods of handling data and combining results of studies, if done, including measures of consistency (e.g., I^2) for each meta-analysis.	p.4
Risk of bias across studies	15	Specify any assessment of risk of bias that may affect the cumulative evidence (e.g., publication bias, selective reporting within studies).	p.4
Additional analyses	16	Describe methods of additional analyses (e.g., sensitivity or subgroup analyses, meta-regression), if done, indicating which were pre-specified.	p.4
**RESULTS**			
Study selection	17	Give numbers of studies screened, assessed for eligibility, and included in the review, with reasons for exclusions at each stage, ideally with a flow diagram.	p.4; Figure 1
Study characteristics	18	For each study, present characteristics for which data were extracted (e.g., study size, PICOS, follow-up period) and provide the citations.	p.4; Table 1
Risk of bias within studies	19	Present data on risk of bias of each study and, if available, any outcome level assessment (see item 12).	p.6; Figure 2, Table S3
Results of individual studies	20	For all outcomes considered (benefits or harms), present, for each study: (a) simple summary data for each intervention group (b) effect estimates and confidence intervals, ideally with a forest plot.	p.6-9; Figure 3; Table 2-3

Synthesis of results	21	Present results of each meta-analysis done, including confidence intervals and measures of consistency.	p.6-9
Risk of bias across studies	22	Present results of any assessment of risk of bias across studies (see Item 15).	p.9-10; Figure 4, Figure S1.
Additional analysis	23	Give results of additional analyses, if done (e.g., sensitivity or subgroup analyses, meta-regression [see Item 16]).	p.10; Figure S1
**DISCUSSION**			
Summary of evidence	24	Summarize the main findings including the strength of evidence for each main outcome; consider their relevance to key groups (e.g., healthcare providers, users, and policy makers).	p.10-12
Limitations	25	Discuss limitations at study and outcome level (e.g., risk of bias), and at review-level (e.g., incomplete retrieval of identified research, reporting bias).	p.12
Conclusions	26	Provide a general interpretation of the results in the context of other evidence, and implications for future research.	p.12
**FUNDING**			
Funding	27	Describe sources of funding for the systematic review and other support (e.g., supply of data); role of funders for the systematic review.	p.12 (Funding: none)

Table S2. Search strategy

Database	Keyword	Date	Results
PubMed	(myocardial infarction OR heart attack OR acute myocardial infarction OR acute coronary syndrome OR STEMI OR NSTEMI OR acute coronary events OR ACS OR ST elevation myocardial infarction OR non-ST elevation myocardial infraction) AND colchicine	2023/01/23	348
Embase			54
Cochrane Library			250

Table S3. Grading of recommendations assessment, development, and evaluation (GRADE) summary of findings.

	Risk of bias	Inconsistency	Indirectness	Imprecision	Publication bias	Certainty
MACE	Not serious	Serious	Not serious	Not serious	Not serious	Moderate
All-cause Mortality	Not serious	Not serious	Not serious	Not serious	Not serious	High
Cardiovascular mortality	Not serious	Not serious	Not serious	Not serious	Not serious	High
Non-fatal Myocardial Infarction	Not serious	Not serious	Not serious	Not serious	Not serious	High
Stroke	Not serious	Not serious	Not serious	Serious	Not serious	Moderate
Angina requiring revascularization	Not serious	Serious	Not serious	Not serious	Not serious	Moderate
Heart failure	Not serious	Not serious	Not serious	Serious	Not serious	Moderate
GI events inclusive all GI symptoms & serious events	Not serious	Serious	Not serious	Not serious	Not serious	Moderate
Hematologic events regardless of severity	Not serious	Not serious	Not serious	Serious	Not serious	Moderate
Infection (regardless of severity)	Not serious	Not serious	Not serious	Not serious	Not serious	High
Diarrhea	Not serious	Serious	Not serious	Not serious	Not serious	Moderate
Thrombosis or embolism	Not serious	Not serious	Not serious	Not serious	Not serious	High
CRP change	Not serious	Serious	Not serious	Serious	Not serious	Low
hsCRP change	Not serious	Serious	Not serious	Serious	Not serious	Low

Table S4. Definitions of MACE for each of the 8 RCTs included in the MACE analysis

Studies	Definition of MACE
Akodad M (2017) [17]	Death, resuscitated cardiac arrest, ventricular arrhythmias, stent thrombosis, myocardial infarction, urgent coronary revascularization and acute heart failure
Akrami M (2021) [11]	Death from any cause, noncardioembolic ischemic stroke, hospital admission due to typical chest pain (STEMI/NSTEMI/unstable angina), urgent need for revascularization and decompensated heart failure
Bouabdallaoui N (2020) [10]	Composite of cardiovascular death, resuscitated cardiac arrest, MI, stroke, or urgent hospitalization for angina requiring coronary revascularization
Bouleti C (2024) [20]	All-cause death, ACS, heart failure events (new hospitalization for heart failure), ischemic strokes, sustained ventricular arrhythmias and acute kidney injury
Jolly SS (2024) [15]	Composite of death from cardiovascular causes, recurrent myocardial infarction, stroke, or unplanned ischemia driven coronary revascularization
Hosseini SH (2022) [35]	Target vessel revascularization, target lesion revascularization, new hospitalization because of heart failure, stroke, nonfatal MI, and cardiac death
Tong DC (2020) [13]	Composite of death from any cause, ACS (STEMI/NSTEMI/unstable angina), ischemia driven urgent revascularization, and noncardioembolic ischemic stroke
Yu M (2024) [34]	Composite of all-cause death, nonfatal myocardial infarction, nonfatal stroke, and revascularization because of ischemia

Table S5. Comparison of MACE definition for each of the 8 RCTs included in the MACE analysis

Studies	All cause death	Cardiovascular death	Non-fatal cardiac arrest	Ventricular arrhythmias	Myocardial infarction (new or recurrent)	Hospitalization due to typical angina (requiring revascularization)	Urgent coronary revascularization	Ischemic stroke	Acute heart failure (with or without hospitalization)	Acute kidney failure	Stent thrombosis	Target vessel/lesion revascularization
Akodad M (2017) [17]	○		○	○	○		○		○		○	
Akrami M (2021) [11]	○					○	○	○	○			
Bouabdallaoui N (2020) [10]		○	○		○	○		○				
Bouleti C (2024) [20]	○			○	○	○		○	○	○		
Jolly SS (2024) [15]		○			○	○		○				
Hosseini SH (2022) [35]		○			○			○	○			○
Tong DC (2020) [13]	○				○	○		○				
Yu M (2024) [34]	○				○	○		○				

Figure S1. Funnel plot analysis of publication bias for MACE.
(p = 0.07; Egger's test)

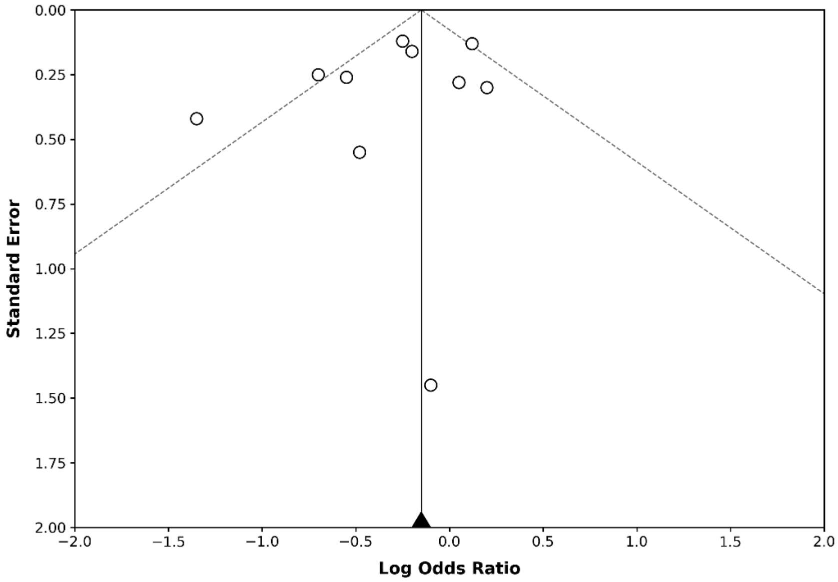

Figure S2. Forest plot showing mean difference (MD) and 95 % CI for CRP (A) and hs-CRP (B).

(A) CRP

	Colchicine			Placebo				Mean Difference
Study or Subgroup	Mean	SD	Total	Mean	SD	Total	Weight	IV, Random, 95% CI
Deftereos S 2015	61.41	10.88	77	40.44	8.48	74	33.4%	20.97 [17.87, 24.07]
Mewton et. al. 2021	12.7	0.04	101	20.6	23.04	91	32.7%	-7.90 [-12.63, -3.17]
Yousefzadeh M2024	20.17	6.66	86	17.28	4.28	86	33.8%	2.89 [1.22, 4.56]
**Total (95% CI)**			**264**			**251**	**100.0%**	**5.40 [-8.70, 19.51]**

Heterogeneity: Tau² = 152.40; Chi² = 135.47, df = 2 (P < 0.00001); I² = 99%
Test for overall effect: Z = 0.75 (P = 0.45)

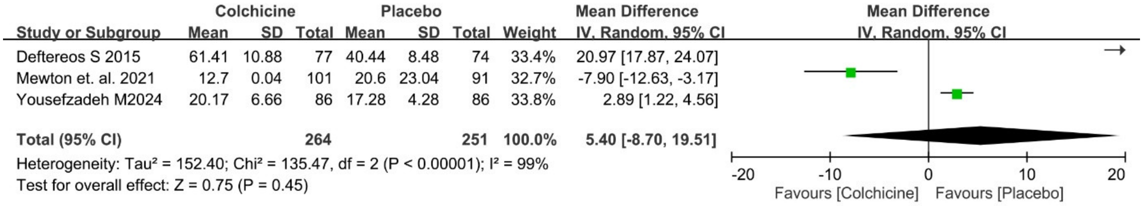

(B) hs-CRP

	Colchicine			Placebo				Mean Difference
Study or Subgroup	Mean	SD	Total	Mean	SD	Total	Weight	IV, Random, 95% CI
Gholoobi, Arash 2021	1.5	0.04	75	0.68	0.02	75	26.6%	0.82 [0.81, 0.83]
Hennessy T 2019	5.3	1.48	111	5.7	2.13	111	24.5%	-0.40 [-0.88, 0.08]
Tardif JC 2019	2.9	2.76	119	3.49	5.75	118	17.9%	-0.59 [-1.74, 0.56]
Wasyanto 2018	3.82	2.2	16	0.57	3.12	16	11.6%	3.25 [1.38, 5.12]
Yu M 2024	0.7	2.96	52	0.5	2.15	52	19.5%	0.20 [-0.79, 1.19]
**Total (95% CI)**			**373**			**372**	**100.0%**	**0.43 [-0.42, 1.28]**

Heterogeneity: Tau² = 0.70; Chi² = 38.30, df = 4 (P < 0.00001); I² = 90%
Test for overall effect: Z = 0.99 (P = 0.32)

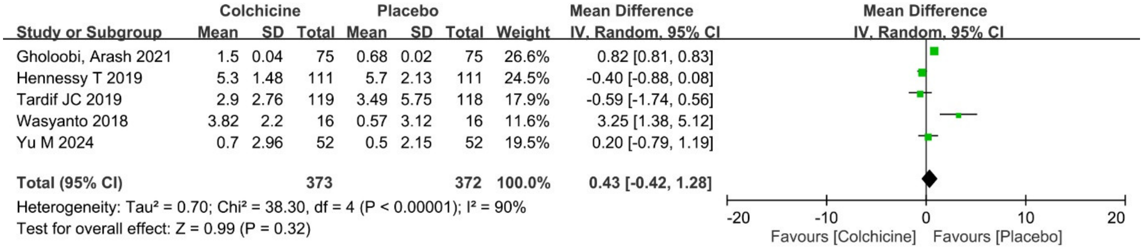

CRP, C-reactive protein; hs-CRP, high-sensitivity CRP. [9,12,14,16,18,19,21,34]
